# A novel loss-of-function *KCNB1* gene variant in a twin with global developmental delay and seizures

**DOI:** 10.3389/fncel.2024.1477989

**Published:** 2024-10-14

**Authors:** Rían W. Manville, Claire L. Illeck, Cesar Santos, Richard Sidlow, Geoffrey W. Abbott

**Affiliations:** ^1^Bioelectricity Laboratory, Department of Physiology and Biophysics, School of Medicine, University of California, Irvine, Irvine, CA, United States; ^2^Valley Children's Hospital, Madera, CA, United States

**Keywords:** DEE, KCNB, KV2.1, neurodevelopmental delay, seizures

## Abstract

Human voltage-gated potassium (Kv) channels are expressed by a 40-member gene family that is essential for normal electrical activity and is closely linked to various excitability disorders. Function-altering sequence variants in the *KCNB1* gene, which encodes the neuronally expressed Kv2.1 channel, are associated with neurodevelopmental disorders including developmental delay with or without epileptic activity. In this study, we describe a 40-month-old fraternal twin who presented with severe neurodevelopmental delay. Electroencephalogram recordings at 19 months of age revealed poor sleep architecture and the presence of multifocal epileptiform discharges. The individual’s fraternal twin was neurotypical, and there was no family history of neurodevelopmental delay or seizures. Whole genome sequencing at 33 months of age for the proband revealed a *de novo* variant in *KCNB1* [c.1154C > T/p.Pro385Leu], encoding a proline-to-leucine substitution at residue 385, in the extracellular region immediately preceding Kv2.1 transmembrane segment 6 (S6). Cellular electrophysiological analysis of the effects of the gene variant in heterologously expressed Kv2.1 demonstrated that homozygous Kv2.1-P385L channels were completely non-functional. Channels generated by a 50/50 expression of wild-type Kv2.1 and Kv2.1-P385L, designed to mimic the proband’s heterozygous status, revealed a partially dominant-negative effect, resulting in an 81% reduction in current magnitude. The dramatic loss of function in Kv2.1 is the most likely cause of the severe developmental delay and seizure activity in the proband, further enriching our phenotypic understanding of *KCNB1* developmental encephalopathies.

## Introduction

Voltage-gated potassium (Kv) channels control electrical excitability in a variety of tissues and cell types. Kv channels contribute to setting the resting membrane potential as well as determining the action potential firing frequency, duration, and morphology in neurons and other excitable cells. Consequently, disruptive gene variants in Kv channels cause a variety of electrical excitability disorders, especially in the brain and the heart. Kv channels form as tetramers of pore-forming *α* subunits each with six transmembrane segments and a pore loop. While most Kv channels can function as a tetramer of α subunits when heterologously expressed, they often co-assemble with ancillary subunits to endow specific properties or cellular locations *in vivo* (McCrossan et al., 2009).

The *KCNB1* gene, located on human chromosome 20q13.3, encodes the α subunit of the Kv2.1 voltage-gated potassium channel. Kv2.1 is widely expressed in mammalian neurons (Bar et al., 2020; Bishop et al., 2015; Veale et al., 2022) and is important for normal neuronal membrane repolarization (de Kovel et al., 2017; Ottschytsch et al., 2002). Pathogenic variants in the *KCNB1* gene can cause a range of human neurodevelopmental disorders, spanning from developmental epileptic encephalopathies (DEEs) to global development delay in the absence or presence of epileptic activity (Bar et al., 2020; Bar et al., 2020). Epileptiform activity linked to *KCNB1* gene variants usually occurs during infancy or childhood and is often pharmacologically intractable. *KCNB1*-associated DEEs can comprise ataxia, behavioral problems, EEG abnormalities, hypotonia, photosensitivity, learning difficulties, and spasticity. Some cases present with atrophy and non-specific periventricular white matter abnormalities detected with MRI (Bar et al., 2020; Bar et al., 2020). In this study, we describe a case of neurodevelopmental delay and seizure activity associated with a *KCNB1* loss-of-function gene variant.

## Case presentation

**Figure 1:**
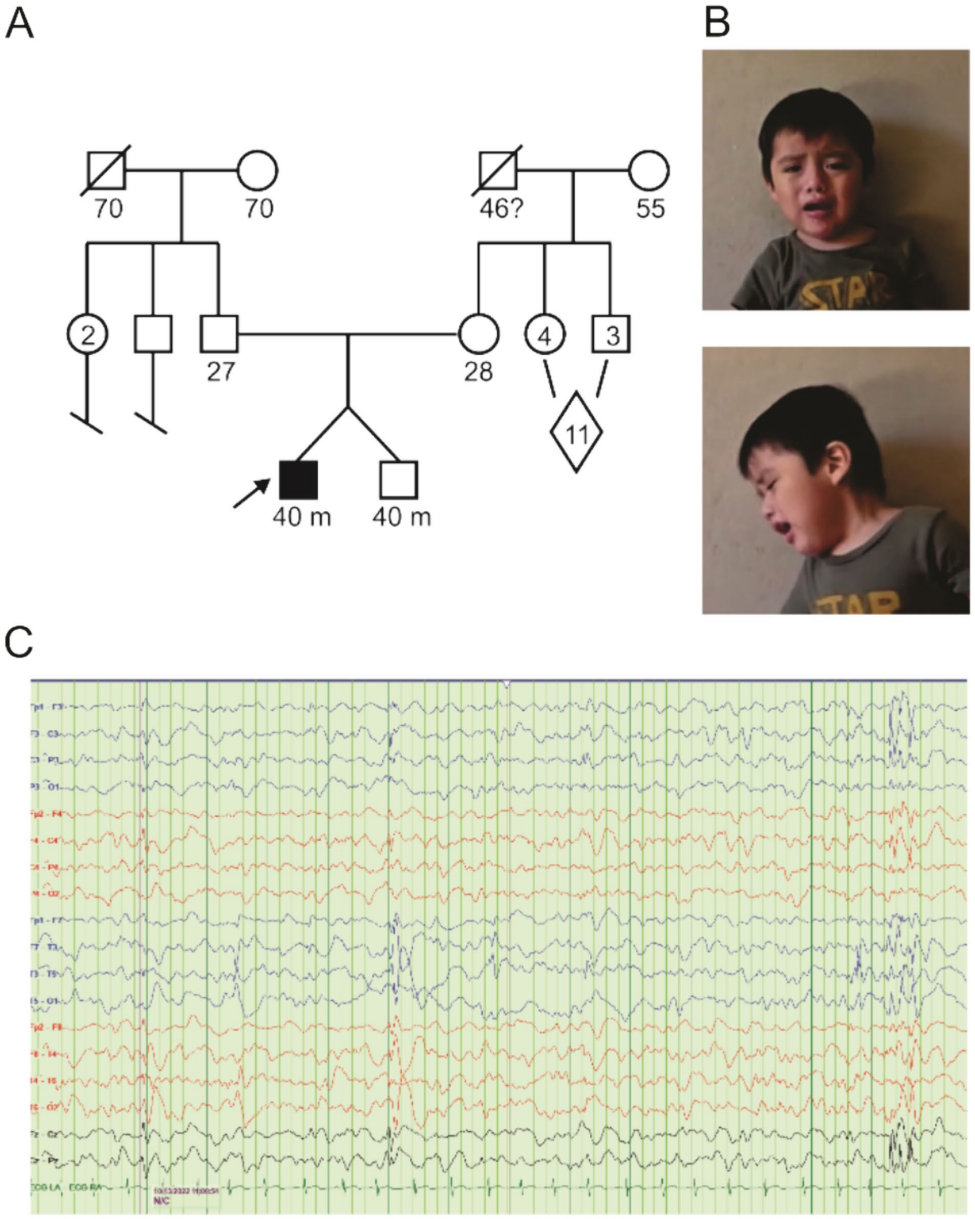
Pedigree and clinical findings. **(A)** Pedigree diagram for KCNB1-P385L in the family under study. The diamond indicates 11 siblings, a mix of male and female. **(B)** Frontal and sagittal images of proband. **(C)** EEG recorded from proband showing focal as well as generalized epileptiform discharges.

The patient is a 40-month-old male child undergoing evaluation for global developmental delay and a possible seizure disorder. He is fraternal twin B, born at 36 weeks of gestation. This is the first pregnancy for his 26-year-old mother, which was uncomplicated, followed by an uncomplicated postpartum course. There is no family history of developmental delays, intellectual disability, seizure disorder, autism, or major behavioral issues (Figure 1A). Both parents are from San Lorenzo, Oaxaca, Mexico. The patient’s birth weight was 6 lb 1 oz (twin A was 6 lb 2 oz), and fetal movements were detected at 24-week gestation for both twins. Fraternal twin A has no physical or developmental issues and was not genetically tested due to a lack of symptomatology. Prior to our patient’s first medical evaluation, he had been receiving physical therapy for 6 months and had just begun occupational therapy. A routine awake and sleep electroencephalogram (EEG) was performed at 17 months of age, which was normal.

At the time of first evaluation at 18 months, the patient had only begun to sit independently and had at most two intelligible words. Positive findings on physical examination were relative macrocephaly, long oval-shaped face, tenting of the upper lip, one café-au-lait macule <1 cm in largest dimension on upper right back just below the neck, occipital plagiocephaly, and forehead prominence with bossing (Figure 1B). His neurologic examination was positive for diminished resting muscular tone (proximally more than distally) with normal muscle bulk. The paucity of spontaneous movement was noted. He was unable to raise either arm above his head. Entities considered in the differential diagnosis at this time included congenital muscular dystrophy and lysosomal disorders—MRI of the brain and screening laboratories including TSH and CK levels were performed and were normal.

A more detailed examination at 19 months of age revealed the following: ability to sit independently but not crawl or pull to stand; walking 6–7 steps with the assistance of a walker; three words; questionable response to name calling; poor visual fixation and following. Audiology, ophthalmology, and genetics evaluations were ordered. Follow-up physical therapy evaluation showed global hypotonia and equivocal Babinski reflexes. At this time, our patient’s mother showed a video of him displaying rhythmic chin movements with upward/left eye deviation for 5 min. The mother also reported “his bottom lip going in and out, eyes rolling back, alternating hand opening and closing and head dropping” on four occasions as well as staring spells accompanied by alternating eye opening and closing and hand stiffening one time or two times daily.

Solid ankle and ankle foot orthotics were prescribed, and a repeat EEG was taken. The EEG (30-min duration, started at 10:10 am), performed at the age of 22 months, revealed poor sleep architecture and the presence of multifocal epileptiform discharges implicating multifocal seizures being present (Figure 1C). Oxcarbazepine was prescribed and titrated upward, achieving control of his seizure activity. At repeat physical therapy evaluation at 28 months, our patient was able to ambulate with frequent falls (he started walking at 26 months). Speech therapy was recommended, and prescriptions for a bath chair, car seat, medical stroller, and soft helmet were written. He underwent right-sided orchiopexy.

Genetics evaluation was performed at 30 months of age. A developmental screen at this time showed severe deficits in all developmental areas—he was non-verbal. Growth parameters were weight 13.9 kg (60th percentile), height 88.2 cm (21st percentile), and head circumference 49.5 cm (55th percentile). Positive findings on physical and neurologic examination were as follows: brachy- and mild turricephaly, prominent forehead with mild bossing, café-au-lait macule as mentioned earlier, mildly ataxic, wide-based aimless gait with purposeless arm movements and mild neck flexion, conjugate but unfocused eye movements, and globally low tone. An ophthalmology referral was made, and a whole genome sequencing (WGS) for the trio was ordered. A repeat physical therapy evaluation at 32 months of age revealed toe walking, poor balance, poor eye contact, hand play, and lack of pointing; an autism evaluation was recommended. Audiology and ophthalmology evaluations were both normal.

**Figure 2:**
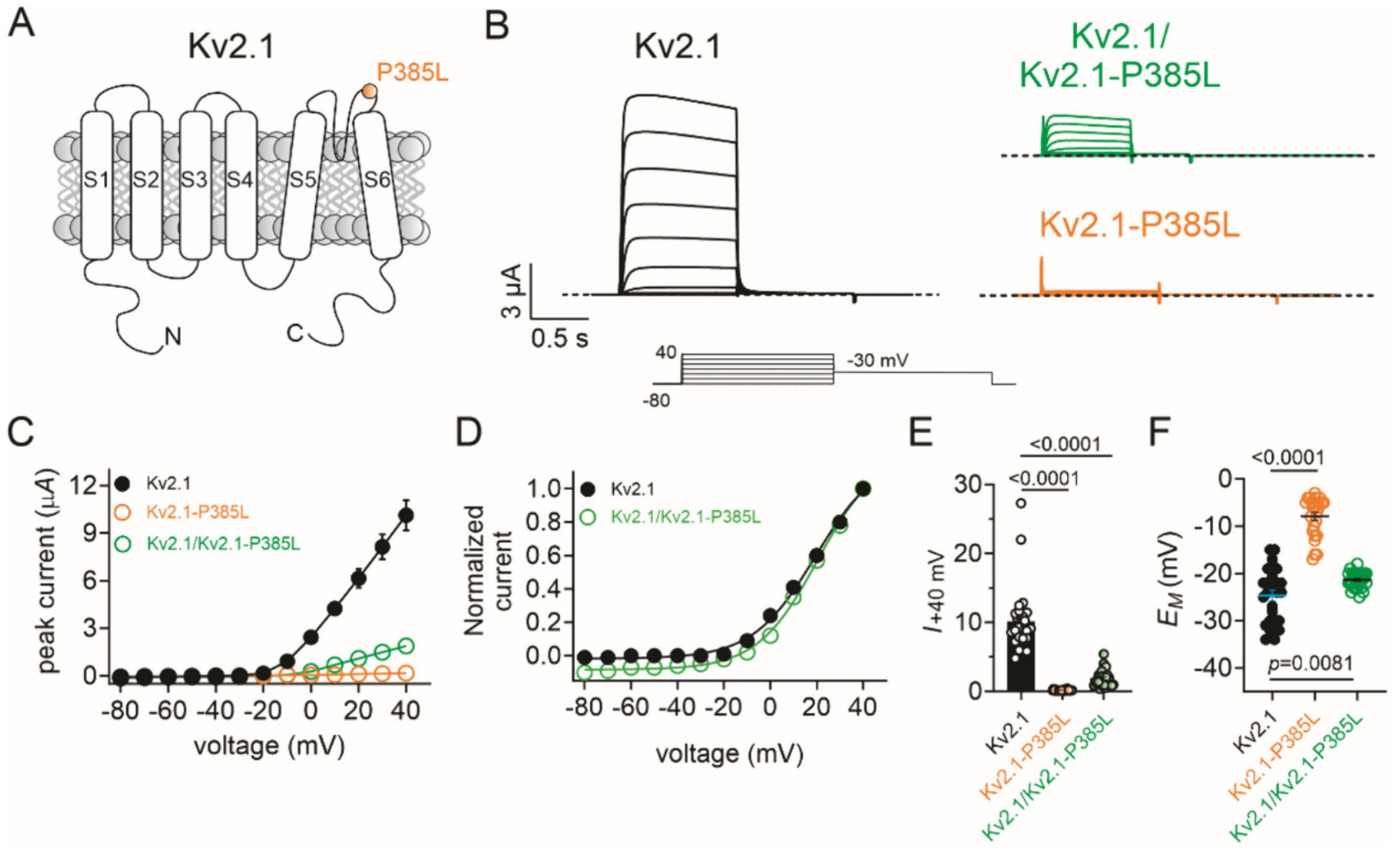
Effects of P385L on Kv2.1 activity. The error bars indicate SEM and when not visible are smaller than the data points. *n* indicates the number of oocytes. Statistical comparisons using the one-way ANOVA. The dashed line indicates the zero current level. **(A)** Transmembrane topology schematic of Kv2.1 showing location of P385L. **(B)** Mean traces for wild-type (Kv2.1), homozygous mutant (Kv2.1-P385L), and heterozygous mutant (Kv2.1/Kv2.1-P385L) channels expressed in oocytes. Scale bars lower left inset; voltage protocol lower inset; *n* = 25–28 per group. **(C)** Mean peak current for traces as in **B**; *n* = 25–28 per group. **(D)** Mean normalized peak current from wild-type and heterozygous mutant currents as in C (*n* = 25–28). **(E)** Comparison of mean peak current at +40 mV from traces in **C** (*n* = 25–28). **(F)** Mean unclamped oocyte membrane potential for oocytes as in **B**; *n* = 25–28 per group.

A follow-up with genetics was conducted at 33 months of age to discuss the results of the WGS, which revealed a *de novo* variant in *KCNB1* [c.1154C > T/p.Pro385Leu], which encodes a proline-to-leucine substitution at residue 385, in the extracellular region immediately preceding transmembrane segment 6 (S6). Sequencing revealed that the proband’s parents were negative for the c.1154C variant (Figures 1A, 2A). The P385L variant is absent from the gnomAD database and is listed as a variant of unknown significance (VUS) in ClinVar - however, assessment in Varsome and Franklin by Genoox variant curation tools rated this variant likely pathogenic. A homozygous VUS in the gene encoding Protein Phosphatase 1, regulatory subunit 21 (PPP1R21), was identified in the proband but was discounted due to dissimilarity to the phenotype associated with PPP1R21-related autosomal recessive neurodevelopmental disorders. Specifically, our patient’s facial gestalt was inconsistent with PPP1R21-related neurodevelopmental disorder nor did he manifest anatomic brain, cardiac, or pancreatic abnormalities, hepatosplenomegaly, optic nerve atrophy, rotatory nystagmus, or metabolic instability.

## Functional characterization of Kv2.1-P385L potassium channels

Pathogenic variants in the *KCNB1* gene, which encodes the Kv2.1 voltage-gated potassium channel, are causative of autosomal dominant developmental and epileptic encephalopathy 26 (DEE 26). The phenotype is characterized by multifocal seizures, marked motor/speech delays, along with other developmental delays (Bar et al., 2020; Bar et al., 2020). A different change in amino acid at position 385 (P385T) has been identified as pathogenic (de Kovel et al., 2017). No gnomAD population frequency for this variant was identified, nor has this variant been previously identified in individuals. Kv2.1-P385L cDNA was constructed (Genscript, Piscataway, NJ) in the pTLNx expression vector, and then from this, we generated cRNA using *in vitro* transcription using the mMessage mMachine SP6 kit (ThermoFisher, Waltham, MA). We injected wild-type Kv2.1 (2 ng), Kv2.1-P385L cRNA (2 ng), or wild-type Kv2.1 + Kv2.1-P385L cRNA (1 ng each) into stage V and VI defolliculated *Xenopus laevis* oocytes. Oocytes were incubated at 16°C for 2 days, and then we recorded currents using a two-electrode voltage clamp (TEVC) and a standard voltage family protocol with a 1-s pulse to depolarizing potentials in 10-mV increments from a −80 mV holding potential, each followed by a 1-s tail pulse to −30 mV. We performed TEVC at room temperature using an OC-725C amplifier (Warner Instruments, Hamden, CT, USA) and pClamp10 software (Molecular Devices, Sunnyvale, CA, USA) 24 h after cRNA injection. The extracellular bath solution contained (in mM): 96 NaCl, 4 KCl, 1 MgCl_2_, 0.3 CaCl_2_, and 10 HEPES, adjusted to pH 7.6 with Tris base. Pipettes (1–2 MΩ resistance) were filled with 3 M KCl.

The wild-type Kv2.1 generated >10 μA voltage-dependent outward potassium ion currents in response to depolarizing voltage pulses, as expected. In contrast, the homomeric Kv2.1-P385L channels were non-functional. The currents generated by co-injection of equal quantities of wild-type and P385L cRNA generated currents with 19% of the peak current magnitude of wild-type Kv2.1 (Figures 2B,C) without changing the voltage dependence of activation (Figure 2D) (data summarized in Figure 2E). Accordingly, the mean resting membrane potential (*E*_M_) of unclamped oocytes expressing homomeric Kv2.1-P385L was +17 mV depolarized compared to that of oocytes expressing homomeric wild-type Kv2.1; the heterozygous channels set the mean resting membrane potential of oocytes at 3 mV more positive than oocytes expressing the homomeric wild-type Kv2.1 (Figure 2F).

**Figure 3:**
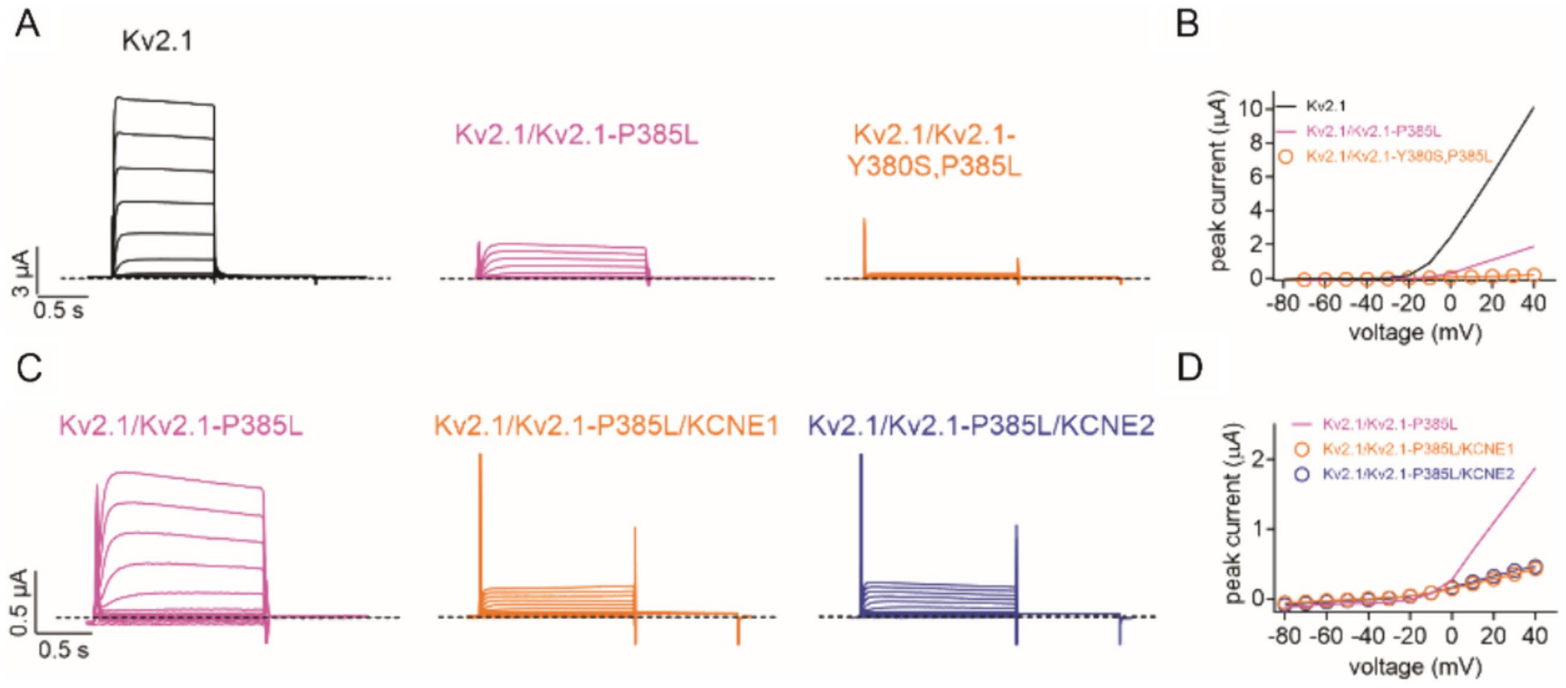
Kv2.1-P385L participates in active channel complexes. **(A)** Representative traces for wild-type (Kv2.1) (*n* = 26), heterozygous mutant (Kv2.1/Kv2.1-P385L) (*n* = 28), and heterozygous double mutant (Kv2.1/Kv2.1-Y380S, P385L) (*n* = 14) channels expressed in oocytes. Scale bars lower left inset; voltage protocol as in Figure 2B. **(B)** Mean peak current for traces as in **A**; *n* values 14–28 as in panel **A**. Lines lacking data points are best-fit lines from Figure 2C. **(C)** Representative traces for heterozygous mutant (Kv2.1/Kv2.1-P385L) alone (*n* = 28) and with KCNE1 (*n* = 36) or KCNE2 (*n* = 34) co-expression in oocytes. Scale bars lower left inset; voltage protocol as in Figure 2B. **(D)** Mean peak current for traces as in **C**; *n* = 28–36 as in panel **C**. Line lacking data points is a best-fit line from Figure 2C.

To test the hypothesis that Kv2.1-P385L participates in active channels at the plasma membrane, we introduced a Y380S mutation into the GYG selectivity filter motif of Kv2.1-P385L to dominant-negatively disrupt channel function of plasma membrane-expressed channels. We found that a 50/50 co-expression of wild-type Kv2.1 with Kv2.1-P385L,Y380S resulted in a complete loss of current (Figures 3A,B). This supports the conclusion that Kv2.1-P385L traffics to the membrane and participates in active K^+^ channels in complexes with wild-type Kv2.1; otherwise, the addition of the Y380S mutation would have had no effect on the current magnitude.

**Figure 4:**
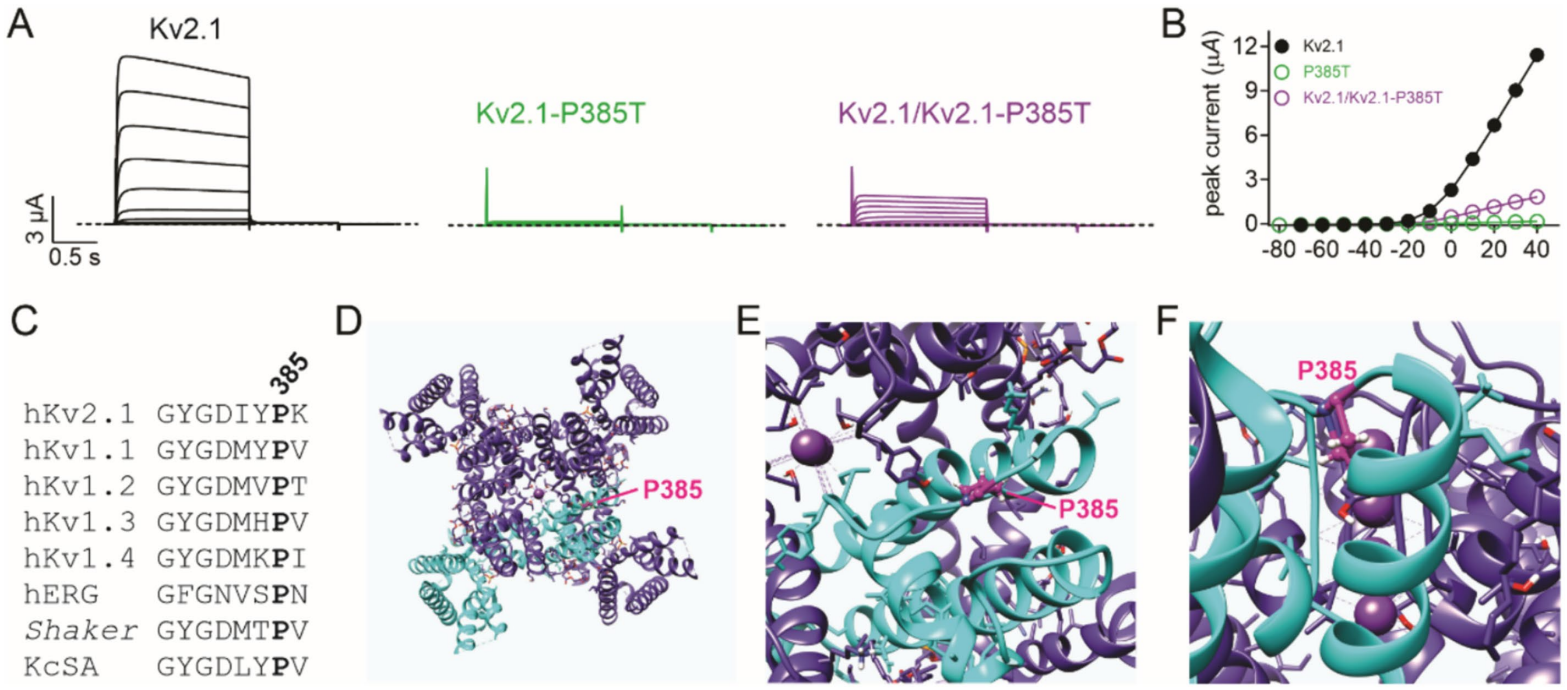
P385 in the context of the Kv2.1 high-resolution structure. **(A)** Mean traces for wild-type (Kv2.1), homozygous, and heterozygous mutant Kv2.1-P385T channels expressed in oocytes. Scale bars lower left inset; voltage protocol as in Figure 2B; *n* = 14–15 per group. **(B)** Mean peak current for traces as in **A**; *n* = 14–15 per group. The error bars indicate SEM, not visible because smaller than the data points. **(C)** Alignment of K^+^ selectivity filter sequence and following residues showing conservation of Kv2.1-P385 among some prominent K^+^ channels. **(D)** An extracellular view of the rat Kv2.1 structure showing one subunit of the tetramer highlighted in cyan, and the P385 equivalent (human numbering) shown in magenta. **(E)** Close-up view from panel **D**. **(F)** Side, close-up view of the rat Kv2.1 pore showing the location of the P385 equivalent (magenta). All images in this figure were plotted from the cryoEM structure of rat Kv2.1 in nanodiscs (PDB: 8SD3) (Fernández-Mariño et al., 2023).

We previously showed that KCNE1 (MinK) and KCNE2 (MiRP1) form complexes with and regulate Kv2.1 (McCrossan et al., 2009). In this study, we found that the co-expression of KCNE1 or KCNE2 further diminished currents formed by wild-type and P385L Kv2.1 (Figures 3C,D). Finally, a previous report indicated that a different substitution at the same position in Kv2.1 (P385T) was associated with a more severe clinical presentation than what we observed for P385L (de Kovel et al., 2017). We tested the effects of the P385T mutation and found that when co-expressed 50/50 with wild-type Kv2.1, there was a similar reduction in current (Figures 4A,B) as we had observed for P385L (Figure 2C).

## Discussion

Kv2.1, encoded by *KCNB1*, is essential for normal somatodendritic excitability in hippocampal and pyramidal neurons (Bekkers, 2000; Du et al., 1998; Korngreen and Sakmann, 2000) and is also important for neurodevelopment; *Kcnb1* null mice, for example, exhibit impaired neuronal migration of neocortical glutamatergic neurons and migratory, synaptic, and morphological defects that persist into adulthood (Bortolami et al., 2023). In this case report, we describe the discovery, clinical manifestations, and functional effects on cellular electrophysiology of a novel pathogenic *KCNB1* mutation (p.P385L), which is a highly conserved residue (Figure 4C) in the extracellular, pore-proximal region just upstream of Kv2.1 S6 (Figures 4D–F). Of the almost 60 previously described *KCNB1* channelopathy patients, 85% had developed epilepsy and all had developmental delays, with varying degrees of severity (Bar et al., 2020; de Kovel et al., 2017; Bar et al., 2020; Xiong et al., 2021). The patient we describe therefore fits well into the known *KCNB1* DEE phenotype.

Previously described pathogenic *KCNB1* gene variants are located throughout the Kv2.1 channel architecture (Bar et al., 2020; de Kovel et al., 2017; Bar et al., 2020; Xiong et al., 2021), from the N-terminal cytoplasmic region (Manville et al., 2024) through to the cytoplasmic C-terminal region (Bar et al., 2020), and the majority are *de novo* as in the case of the variant we describe (Bar et al., 2020; de Kovel et al., 2017; Bar et al., 2020; Xiong et al., 2021). As expected, there is a particular clustering of pathogenic variants in and around the Kv2.1 voltage sensor and the pore, as in the case of P385L. Another pathogenic variant at this position, P385T, was reported by de Kovel et al. (2017). While P385L was classified at the time of writing as a VUS on ClinVar, P385T is classified as pathogenic (ClinVar) or likely pathogenic (Saphetor). The P385T variant is much more severe clinically than the P385L we report herein; the P385T variant is associated with focal dyscognitive, drop attacks, tonic, nocturnal tonic, clonic seizures, and myoclonus, with near continuous epileptiform activity on EEG at the age of 16. The P385T-associated developmental delay was also classified as severe, with the patient (male, at the time of publication was 17 years old) being non-verbal; walking only with assistance, with an ataxic gait, having autism, exhibiting scoliosis, and requiring gastric tube feeding (de Kovel et al., 2017). The physicochemical properties of the threonine versus leucine side chains suggested a possible mechanism for the increased severity of disease; while leucine and proline are hydrophobic amino acids, threonine is more polar. P385 is located in the extracellularly exposed linker region between the Kv2.1 pore loop and S6 (Figures 1A, 4D,F), and the proline side chain points inward toward the channel and membrane interior (Fernández-Mariño et al., 2023), placing it in proximity to other side chains and/or membrane lipids (Figure 4F), with which it may need to interact for normal channel function. However, the P385T mutation had similar effects to P385L when tested herein. Other interacting proteins may dictate the severity of the mutation’s effect, or differences in the genetic background of the patients could play a role. For instance, we found that co-expression of KCNE1 or KCNE2 exacerbated the loss of current caused by the P385L variant, demonstrating that interacting proteins can modulate the phenotypic outcomes of variants at P385.

The results of introducing a selectivity filter mutation into the Kv2.1-P385L subunit were consistent with Kv2.1-P385L reaching the membrane and participating in active channels (Figures 3A,B). Assuming that P385L subunits co-assemble normally with wild-type Kv2.1 channels, the binomial distribution of two equally expressed subunit types within a tetrameric channel structure suggests that the 81% reduction in current magnitude observed in the heterozygous condition is approximately consistent with channels in cellular electrophysiology experiments tolerating only one P385L subunit per tetramer. In this case, only 5 out of 16 fully formed channel proteins would be predicted to have 0 or 1 P385L subunits within the tetramer, while the remainder would contain 2 to 4 P385L subunits. This partial dominant negative effect is consistent with the importance of residues in and around the pore region and with the high degree of conservation of the proline at position 385 among other Kv channels (Frech et al., 1989).

In summary, we describe the clinical phenotype and cellular electrophysiological effects of a novel substitution, Kv2.1-P835L, which greatly impairs channel function. The substation is encoded by a *de novo* variant (c.1154C > T) and is associated with a developmental encephalopathy encompassing neurodevelopmental delay and seizures, adding to our understanding of *KCNB1* DEEs and their clinical spectrum.

## Data Availability

The raw data supporting the conclusions of this article are available at doi:10.5061/dryad.jq2bvq8k2.
